# Genotype-by-Inhibitor Interactions to Dissect Enterovirus Replication

**DOI:** 10.21203/rs.3.rs-7660613/v1

**Published:** 2025-09-29

**Authors:** William Bakhache, Walker Symonds-Orr, Patrick T. Dolan

**Affiliations:** 1 Quantitative Virology and Evolution Unit, Laboratory of Viral Diseases, NIH-NIAID Division of Intramural Research, Bethesda, MD, USA

**Keywords:** Positive-sense RNA viruses, Enteroviruses, Replication organelles, Deep mutational scanning, Pharmacological perturbations, Structural modeling, Virus–host interactions

## Abstract

Replication organelles of positive-sense RNA viruses are essential to virus biology, yet their molecular mechanisms remain poorly defined. While deep mutational scanning (DMS) measures the impact of mutations across viral proteins, it cannot resolve their effects on specific functions. Here, we present a strategy to integrate mutational scanning in the context of specific virus- and host-targeted inhibitors with structural modeling to dissect structural and mechanistic details of *Enterovirus* replication. Our results reveal key insights into the function of nonstructural proteins in the context of viral replication. We use this approach to clarify the modular architecture of the viral 2C protein, dissecting the functional partitioning of its ‘virus-facing’ cytoplasmic, enzymatic domain from ‘host-facing’ functions at membrane-binding domain, revealing evidence for computationally-predicted structural transitions associated with host protein binding. We further show that inhibition of the 3C protease enriches for mutations in 2A, highlighting compensatory crosstalk between viral proteases. Finally, targeting host phospholipid synthesis triggers a dose-dependent shift in mutational tolerance in the viral 3A protein, showing how preference for distinct interaction interfaces with either a host enzyme or its adapter protein varies across inhibitory environments. Our approach, which quantifies the impact of pharmacological probes on viral fitness by comprehensive mutational scanning, creates a virtuous cycle where DMS validates and refines structural predictions that, in turn, serve to contextualize mutational data, all toward a more complete model of positive-sense RNA virus replication.

## Graphical Abstract

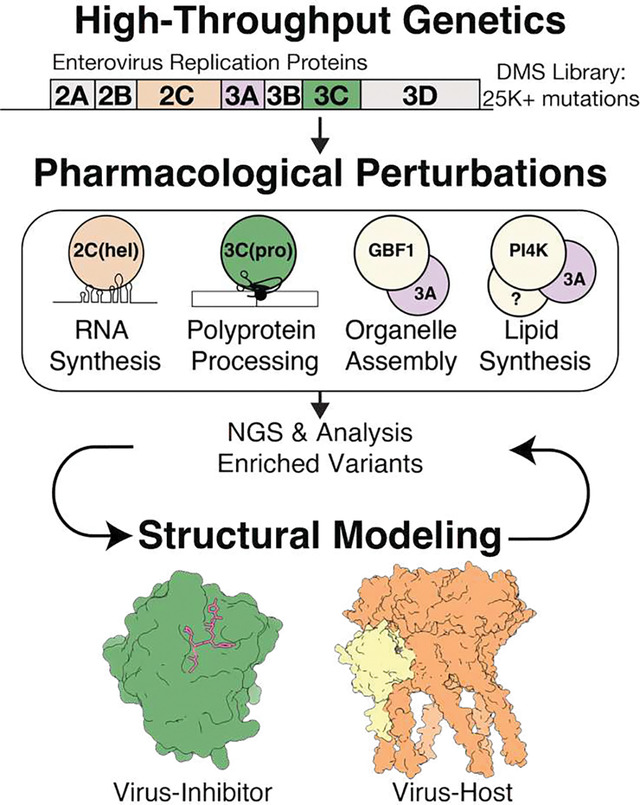

## INTRODUCTION

Like all positive-sense RNA viruses, the replication complex (RC) of *Enteroviruses* brings together viral and host factors to reorganize host lipids and organelle structures for the production of viral genomes (1). The *Enterovirus* genus includes a diverse group *of* human pathogens, such as Enterovirus A71 (EV-A71), Coxsackievirus B3, and poliovirus (2), making their replication machinery a key therapeutic target (3). Although prior work has identified the viral and host factors involved, the molecular details of *Enterovirus* RC formation remain poorly understood. This complexity presents a challenge for antiviral targeting; a deeper mechanistic understanding could therefore reveal new vulnerabilities for therapeutic development.

The *Enterovirus* replication proteins (2A-2C, 3A-3D) perform the diverse enzymatic and membrane-binding functions required to orchestrate RC formation (4). Their essential roles have made them attractive targets for small-molecule inhibitors (3) including direct-acting inhibitors targeting viral enzymes like the 3C protease (e.g., Rupintrivir) (5) and 2C ATPase/helicase (e.g., Guanidine hydrochloride) (6), as well as host-targeted inhibitors against essential factors like GBF1 (e.g., Brefeldin A) (7) and PI4KIIIβ (e.g., Enviroxime) (8). Most of these compounds show limited efficacy *in vivo*, either due to the rapid emergence of resistance or pharmacological limitations (3). Nevertheless, these inhibitors provide a valuable toolkit to probe viral and host functions during replication.

Deep mutational scanning (DMS) quantifies the effects of all possible mutations in viral proteins, robustly identifying residues critical for virus replication (9–11). However, standard DMS experiments measure mutational tolerance that is shaped by an aggregate of all functional constraints, making it difficult to distinguish specific protein functions. This limits the use of DMS in informing mechanistic models of virus replication.

Here, we present an integrated strategy that combines DMS with targeted pharmacological perturbations and structural modeling to overcome this limitation. We apply DMS on the replication proteins of the prototypical and clinically relevant *Enterovirus* EV-A71 in the presence of a panel of well-characterized viral- and host-targeted inhibitors. This approach allowed us to functionally segregate viral protein domains, uncover compensatory enzymatic mechanisms, and map the interfaces of virus-host interactions. Our findings provide molecular details for the assembly of *Enterovirus* RCs and establishes a powerful framework for dissecting positive-sense RNA virus replication.

## RESULTS

### Using Inhibitors to Map the Functional Landscape of the EV-A71 Replication Complex

**Figure 1:**
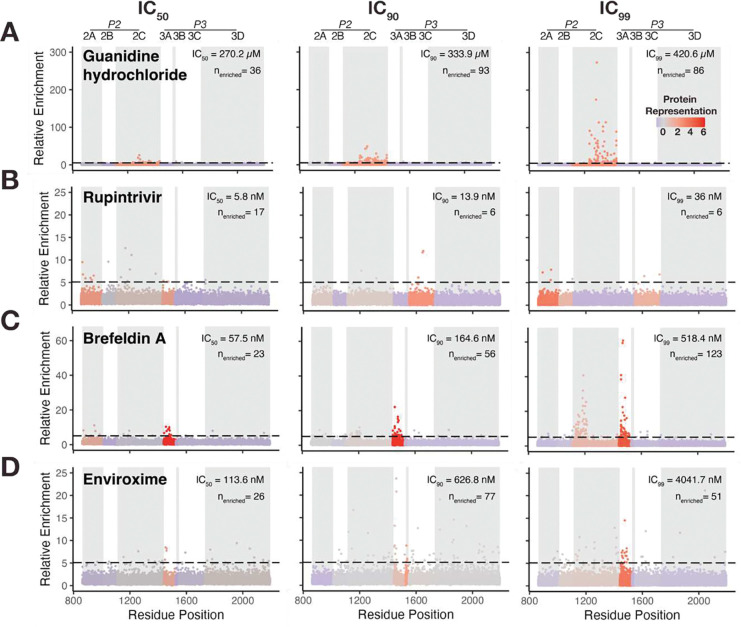
Deep Mutational Scanning in Combination with Inhibitors Probes Functions of Replication Proteins Manhattan plots showing the enriched variants at IC_50_, IC_90_, IC_99_ for **(A)** Guanidine hydrochloride, **(B)** Rupintrivir, **(C)** Brefeldin A, and **(D)** Enviroxime. Data points are colored according to the viral protein representation of enriched mutations. The black dashed line is the enrichment score threshold above which a variant is considered enriched. n_enriched_ represents the total number of detected enriched mutations for each condition.

To functionally map the EV-A71 RC, we applied DMS on the EV-A71 replication proteins (10), under selective pressure from four distinct replication inhibitors (Supplementary Figure 1). The DMS virus population was propagated for a single round of infection at three inhibitory concentrations (IC50, IC90, IC99) for each compound (Supplementary Figure 1). For each inhibitor, we calculated the relative enrichment of every variant compared to the control condition, generating a unique enrichment profile that corresponds to the compound’s mechanism of action (Figure 1A–D). This approach transforms each inhibitor into a molecular probe, allowing us to define the functional dependencies within the viral RC.

As a proof-of-concept, we first used the well-characterized Guanidine hydrochloride, which restricts *Enterovirus* replication by inhibiting the enzymatic activities of the 2C ATPase/helicase (12). Consistent with its known target, selection with this inhibitor enriches for mutations (n=215) exclusively within 2C (Figure 1A), validating our approach of combining DMS with pharmacological manipulation to identify functional sites of viral proteins.

Next, we used Rupintrivir, a potent inhibitor of the 3C protease (5, 13), to probe its functional constraints. This selection resulted in only 29 enriched mutations, supporting that Rupintrivir targets a functional site with limited tolerance for mutations. While mutations were enriched in 3C (Figure 1B), we also observed an unexpected enrichment of mutations in the other viral protease, 2A. This novel finding points to an uncharacterized functional dependency between the two viral proteases, suggesting that mutations in 2A may compensate for impaired 3C activity.

To explore interactions with host factors, we used Brefeldin A which inhibits *Enterovirus* replication by stabilizing an inactive form of the host factor Arf1 (Arf1-GDP) through interactions with its guanine nucleotide exchange factor (GEF) GBF1 (14–16). Consistent with the role of the viral 3A protein in recruiting GBF1 (17), the mutational signature for Brefeldin A was predominantly located within 3A (Figure 1C). At the IC99 condition, mutations also emerge in 2C suggesting a functional link between 3A-host factor interactions and the 2C protein.

Finally, we used Enviroxime to probe the viral machinery that interacts with the PI4KIIIβ host factor, an enzyme responsible for PI4P phospholipid biogenesis at RCs. Selection for Enviroxime leads to enrichment of mutations in 3A (Figure 1D), consistent with its role in recruiting PI4KIIIβ (18–20). Beyond the primary enrichment in 3A, we also observe significant mutational enrichment across other replication proteins, including 2C and 3D. These results provide genetic evidence that PI4P-enriched membranes modulate multiple components of the viral replication machinery, as has been previously suggested (21, 22).

### Rupintrivir Reveals Compensatory Protease Functions

**Figure 2:**
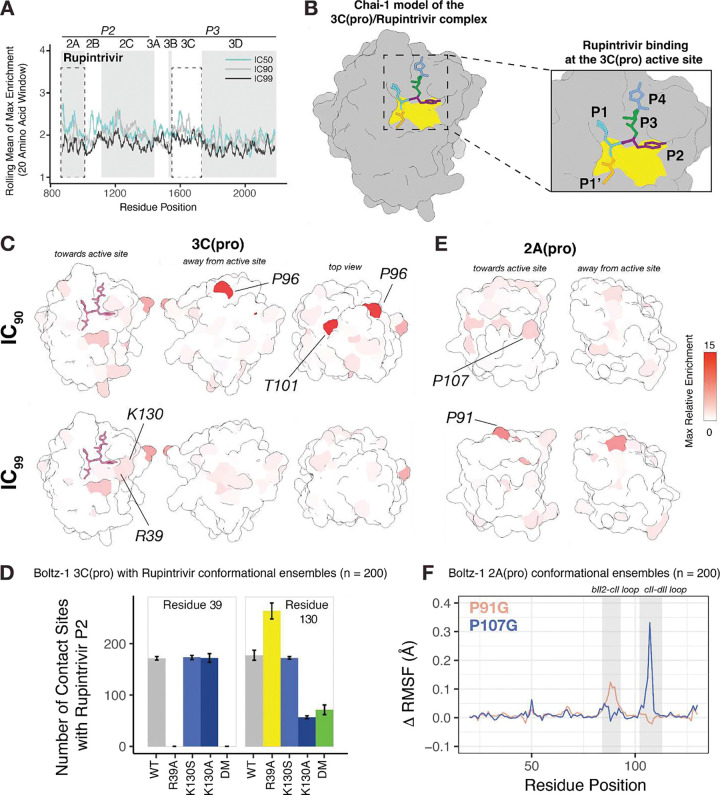
Rupintrivir Inhibition Dissects Viral Protease Functions (A) Line plot showing the max enrichment of Rupintrivir enriched mutations using a 20 amino acid sliding window across the EV-A71 replication proteins. (B) Molecular model (Chai-1) of the 3C protease bound to Rupintrivir. (C) Structural mapping of Rupintrivir enriched mutations (at IC_90_ and IC_99_) on the 3C protease (Chai-1) with Rupintrivir shown in pink. (D) Bar graph showing the mean number of contact sites between the amino acid residues 39 and 130 and the Fluorine atom at the P2 position in Rupintrivir. Error bars represent the standard deviation from three independent Boltz-1 runs. DM represents the double mutant with both mutations R39A and K130A. (E) Structural mapping of enriched Rupintrivir mutations (at IC_90_ and IC_99_) on the 2A protease (AlphaFold3). (F) Difference in Root Mean Square Fluctuation (Δ RMSF) between mutant and wild-type 2A protease conformational ensembles, as predicted by Boltz-1. n represents the number of diffusion samples generated during the prediction.

To understand the functions of viral proteases, we focused on the mutational profile upon Rupintrivir treatment. This profile shows no distinct enrichment sites across the viral replication proteins (Figure 2A); instead, the few enriched mutations were localized specifically within the two viral proteases, 2A and 3C (Figure 1B). The specificity of this inhibitor makes it a useful tool to study the functions of these proteases.

To clarify the structural context of enriched mutations, we used the Chai-1 structure prediction platform to resolve the interaction between Rupintrivir and the 3C protease. Chai-1 predicts the docking of Rupintrivir at the 3C active site, consistent with a published structure of this complex (23) (Figure 2B). Mapping the enrichment scores at IC_90_ on the 3C-Rupintrivir complex shows that most enriched mutations are allosteric located at residues P96 and T101 (Figure 2C). At the higher IC_99_ concentration, moderately enriched mutations appear in residues R39 and K130, which directly contact the Fluorine atom at the P2 position of Rupintrivir (23–25). We hypothesized that these mutations could disrupt Rupintrivir binding by altering contact sites with its P2 moiety.

To test this hypothesis, we generated conformational ensembles of the wild-type or mutant 3C protease bound to Rupintrivir using the Boltz-1 platform. Our wild-type ensemble of the 3C-Rupintrivir complex confirms that R39 and K130 are the primary residues contacting the inhibitor’s P2 Fluorine atom (Figure 2D). The R39A mutation completely abolishes contacts at this position. At position 130, the enriched mutations have varied effects; the K130A substitution causes a threefold reduction in contacts, whereas the K130S substitution shows no significant change in contacts. As expected, the double mutant (R39A/K130A) exhibits a decrease in contact at both residues, effectively disrupting this binding pocket. These changes did not alter the flexibility of the generated 3C protease conformational ensembles. (Supplementary Figure 2A–D). However, the disruption of contact sites is associated with a significant shift in the Coulombic electrostatic potential of the P2 binding pocket towards a more negative charge, possibly impacting Rupintrivir affinity for the 3C protease (Supplementary Figure 2E).

We next investigated the unexpected enrichment of mutations in the 2A protease. In contrast to the allosteric sites in 3C, enriched mutations in the 2A protease center around the active site and were amino acid changes from proline to glycine residues within the bII2-cII and cII-dII loops (Figure 2E). Based on their nature and location, we hypothesized that these mutations might alter loop flexibility. To test this hypothesis, we generated ensembles of the wild-type or mutant 2A protease using Boltz-1 with a low step scale to enhance structural sampling (Figure 2F and Supplementary Figure 2F, G). Analysis of proline to glycine mutations reveals a consistent increase in Root Mean Square Fluctuation (RMSF) values compared to the wild-type protein. This suggests that these mutations enhance the flexibility of the loops in proximity to the active site, potentially allowing the 2A protease to better accommodate and cleave 3C substrates. While requiring biochemical validation, this model provides a powerful mechanistic hypothesis for the functional compensation observed in our genetic screen.

### Guanidine Hydrochloride and Brefeldin A Distinguish Two Functional Interfaces in 2C

**Figure 3:**
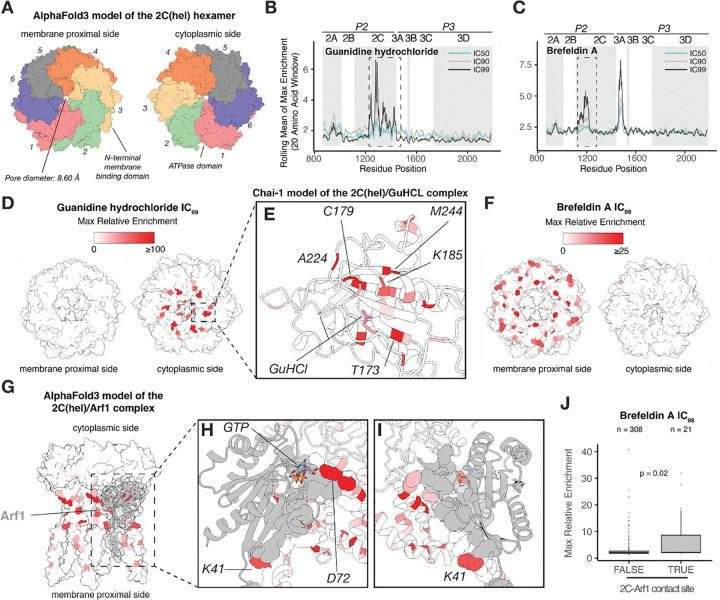
Guanidine hydrochloride and Brefeldin A Treatment Reveals Two Functional Interfaces of the 2C ATPase/helicase **(A)** AlphaFold3 predicted structure of the EV-A71 2C helicase hexamer. Max enrichment of variants to Guanidine hydrochloride **(B)** and Brefeldin A **(C)** across EV-A71 replication proteins (20-amino acid sliding window). **(D)** Localization of Guanidine hydrochloride enriched mutations (IC_99_) on the 2C helicase structure. **(E)** Close-up view of the 2C ATPase domain showing the modeled interaction with Guanidine hydrochloride (Chai-1 model). **(F)** Localization of Brefeldin A enriched mutations (IC_99_) on the 2C helicase structure. **(G)** AlphaFold3 model of the 2C helicase hexamer in complex with the active form of the Arf1 protein. **(H-I)** Detailed views of the 2C-Arf1 interaction sites, with contact residues shown in sphere representation. **(J)** Box plot comparing the max relative enrichment of Brefeldin A mutations at residue positions that interact (TRUE) or do not interact (FALSE) with Arf1. Statistical significance was determined using a one-sided Wilcoxon-Mann-Whitney test.

Our screen identified 2C as a key viral protein in both Guanidine hydrochloride and Brefeldin A inhibitory conditions (Figure 1A, C). *Enterovirus* 2C is a multifunctional protein crucial for viral replication with ATPase-dependent helicase and membrane-binding activities (26). 2C forms a hexameric ring that is required for its ATPase activity (27, 28). To understand the structural context for these distinct pressures, we generated an AlphaFold3 model of the EV-A71 2C hexamer which was consistent with a published structure (29) (Figure 3A, Supplementary Figure 3A).

Guanidine hydrochloride and Brefeldin A treatments show clear dose-dependent patterns within 2C (Figure 3B, C). Guanidine hydrochloride enriched mutations map to the cytoplasmic side, specifically clustering within the ATPase domain (Figure 3D). Chai-1 modeling shows that these mutations were present near the predicted Guanidine hydrochloride binding site (Figure 3E). In addition, the glycine residue 321, located in the α−6 helix of the pocket binding domain, was also a hotspot for mutations (Supplementary Figure 3A). This residue is distant from the active site of the helicase pointing to an allosteric mechanism that alters 2C-2C oligomer interactions, consistent with the link between 2C hexamerization and its ATPase activity (27).

Conversely, Brefeldin A mutations map to the membrane-proximal side of the 2C hexamer (Figure 3F). This spatial segregation highlights the distinct functional domains within 2C probed by these two compounds. Previous research has shown that the poliovirus 2C can interact with Arf1(30). Based on this work, we hypothesized that mutations in EV-A71 2C could favor recruitment of active Arf1 (Arf1-GTP) bypassing the need for GBF1.

To test this hypothesis, we modeled the 2C hexamer-Arf1-GTP interaction using AlphaFold3 (Figure 3G–I). The resulting model predicts Arf1 binding to the membrane-proximal surface of the 2C hexamer, inducing a conformational change in the 2C N-terminal region to accommodate this host factor (Supplementary Figure 3B). Enriched variants K41 and D72 were predicted to directly interact with Arf1, with the role of residue 72 consistent with previous poliovirus work (30). Crucially, the predicted Arf1-interacting surface of 2C shows a significantly higher mutational enrichment than non-interacting regions (p= 0.02, Wilcoxon-Mann-Whitney test), directly linking our experimental DMS data to our structure prediction models (Figure 3J).

### Host-Directed Inhibitors Define the Molecular Interfaces between 3A and Host Factors

**Figure 4:**
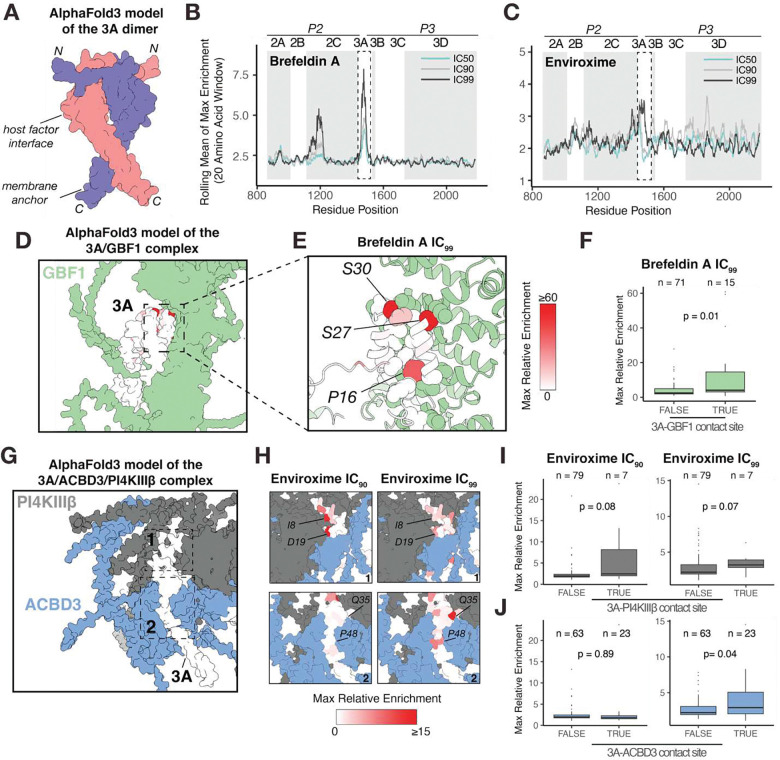
Enviroxime and Brefeldin Dissect Molecular Mechanisms of 3A Interactions with Host Factors **(A)** AlphaFold3 predicted structure of the EV-A71 3A dimer. Max enrichment of variants to Brefeldin A **(B)** and Enviroxime **(C)** across EV-A71 replication proteins (20-amino acid sliding window). **(D)** Alphafold3 model of the 3A dimer with GBF1. **(E)** Detailed view showing the interaction of GBF1 with 3A highlighting the enrichment for Brefeldin A, with contact residues shown in sphere representation. **(F)** Box plot comparing the max relative enrichment of Brefeldin A mutations at residue positions that interact (TRUE) or do not interact (FALSE) with GBF1. Statistical significance was determined using a one-sided Wilcoxon-Mann-Whitney test. **(G)** Alphafold3 model of the 3A dimer/ACBD3/PI4KIIIβ complex. **(H)** Detailed views of the 3A/PI4KIIIβ interaction site and the 3A/ACBD3 interaction mapping Enviroxime enriched variants. Box plot comparing the max relative enrichment of Brefeldin A mutations at residue positions that interact (TRUE) or do not interact (FALSE) with either PI4KIIIβ **(I)** or ACBD3 **(J)** contact sites.

The viral protein 3A forms a homodimer that anchors the RC to membranes recruiting multiple host factors, a characteristic conserved across *Enteroviruses* (17, 20). To investigate 3A enriched mutations to Enviroxime and Brefeldin A, we generated an AlphaFold3 model of the EV-A71 3A homodimer (Figure 4A). Brefeldin A treatment enriches mutations at a single site in 3A (Figure 4B), which contrasts with Enviroxime treatment, where we observe a dose-dependent shift across distinct sites of the protein (Figure 4C).

Brefeldin A mutations emerge mainly in 3A concordant with its role in the recruitment of GBF1 to RCs. Modeling 3A-GBF1 interactions showed that Brefeldin A enriched variants predominantly mapped to predicted contact sites with the host factor (p= 0.01, Wilcoxon-Mann-Whitney test) (Figure 4D–F). These findings provide molecular insights into 3A–GBF1 interactions that have remained inaccessible to structural characterization owing to the large size of GBF1 and the poor solubility of full-length 3A.

Enviroxime targets the host enzyme PI4KIIIβ that is suggested to be recruited indirectly to RCs through interactions between 3A and the adapter protein Acyl-coenzyme A binding domain containing 3 (ACBD3) (31). To clarify 3A’s interaction with these host factors, we modeled a 3A/ACBD3/PI4KIIIβ complex. Our model shows PI4KIIIβ interacting primarily with the 3A N-terminal region, and ACBD3 with 3A’s central region. This structural arrangement aligns with contact residues defined by Hydrogen/Deuterium exchange mass spectrometry between Aichi virus 3A and ACBD3/PI4KIIIβ (32) (Supplementary Figure 4A). IC90 enriched variants localize mainly to the PI4KIIIβ interaction site, while IC99 variants map to the ACBD3 interaction site (Figure 4G–J). In addition, Enviroxime mutations could not be explained by 3A contact sites with GBF1 suggesting that it does not act as the adapter for PI4KIIIβ recruitment (Supplementary Figure 4B–C). This dose-dependent mutational shift indicates a switch in viral strategy for overcoming PI4KIIIβ inhibition. We propose that at lower inhibitory concentrations, minor alterations to the direct 3A-PI4KIIIβ enzyme interface are sufficient to overcome inhibition. However, at higher concentrations, a remodeling of the 3A-ACBD3 interaction interface becomes necessary.

## DISCUSSION

A hallmark of positive-sense RNA viruses is their remodeling of host membranes to form RCs for viral genome replication. While RC structures are well-resolved in some genera, such as *Alphaviruses* (33–36)*,* they remain poorly defined in *Enteroviruses*. Moreover, structural information must be integrated with functional data to dissect RC mechanisms. Our work takes a unique approach combining high-throughput genetics to define functional constraints, pharmacological inhibitors to probe specific replication steps, and structural modeling to reveal molecular details of the *Enterovirus* replication machinery. This strategy uncovers the segregation of functions within viral proteins, compensatory roles of viral proteases, and virus–host interaction interfaces.

Probing for protease functions using the 3C inhibitor, Rupintrivir, showed compensatory mutations in 2A, suggesting that this protease can functionally replace 3C activities, possibly through changes in its structural dynamics as suggested by our analysis. Notably, the 2A proteases of *Picornaviridae* display extensive functional diversity, with five distinct classifications (37), which may explain this compensatory mechanism.

Our structural and functional analysis of the viral protein 2C segregates its functions, with the cytoplasmic domain mediating ATPase/helicase activity and the membrane-proximal domain engaging the host factor Arf1. This 2C-Arf1 interaction stands as an alternative to the canonical 3A-GBF1-Arf1 pathway highlighting a redundancy in Arf1 recruitment strategies. Our work suggests that ancestral *Enteroviruses* possessed both Arf1 recruitment modes, as only a few mutations are needed to switch between the different recruitment strategies (this study and (30)). The conservation of an Arf1 interaction motif supports the ancient origin of this interaction, especially considering Arf1’s fundamental role in eukaryotic organelle biogenesis, tracing back to Asgardian archaea (38).

This study also helps to clarify conflicting reports on the recruitment of the essential host lipid kinase PI4KIIIβ. Our data support a model where PI4KIIIβ is recruited to replication organelles through a complex formed between the viral protein 3A and the host adapter protein ACBD3, independent of the host factor GBF1. Furthermore, the dose-dependent effects of Enviroxime at the 3A–ACBD3 and 3A–PI4KIIIβ interfaces suggest a hierarchical engagement of these host factors, modulated by PI4KIIIβ activity.

This work provides key insights into the replication machinery of *Enteroviruses*. Incorporating a broader range of pharmacological probes and extending to other *Enteroviruses* will further detail the conserved and divergent replication mechanisms across this genus and guide the development of pan-*Enterovirus* antivirals.

Future work can build on this framework by performing higher order DMS approaches to map the interaction interfaces between viral proteins and essential host factors. Furthermore, integrating long-read sequencing technologies allow access to the genomic context of mutations, revealing how these interactions are coordinated. Ultimately, this functional framework will need to be integrated with existing structural, biochemical, and systems-level studies for a more complete understanding of positive-sense RNA virus replication.

## MATERIALS AND METHODS

### Cells and Reagents

RD (ATCC, CCL-136) were cultured in Dulbecco’s Modified Eagle Medium (DMEM) (ATCC, 30–2002) with 10% Fetal Bovine Serum (FBS) (Gibco, 10437–028) and maintained at 37°C with 5% CO2. The FBS concentration was lowered to 5% during infection experiments. The inhibitors were purchased from Sigma-Aldrich and include Rupintrivir (PZ0315), Guanidine hydrochloride (G7294), Enviroxime (SML2995), and Brefeldin A (B7651).

### Inhibitor characterization

EV-A71 (Tainan/4643/98 strain) was used to infect 20,000 RD cells at a low inoculum (30ul) at an MOI of 0.1 for 1 hr in a 96-well plate. After the incubation period, the inoculum was replaced with media containing different concentrations of inhibitors or vehicle control. After 16 hrs, RNA was extracted and used for qPCR experiments targeting the 5’ UTR of the virus or the GAPDH gene using the Luna Cell Ready One-Step RT-qPCR Kit (NEB, E3030S). Primers for amplifying the 5’UTR region of the virus were GCCCCTGAATGCGGCTAATC (forward) and GGACACCCAAAGTAGTCGGTTC (reverse). Primers used for the GAPDH region were GTCTCCTCTGACTTCAACAGCG (forward) and ACCACCCTGTTGCTGTAGCCAA (reverse).

The CellTiter-Glo 2.0 Cell Viability Assay (Promega, G9242), a luminescent-based assay that quantifies ATP levels, was used to evaluate the impact of the different inhibitors concentrations on cell viability in the same conditions described above.

### Virus passaging with inhibitors

Deep mutational scanning (amino acid substitution libraries) of the EV-A71 replication proteins, previously described in our work on EV-A71 mutational tolerance (10), was used. Passage 1 virus was passaged for 24 hours in a T75 flask (6 million RD cells) at MOI 0.1 with IC_50_, IC_90_, or IC_99_ of the respective inhibitors or control condition. The virus was freeze-thawed two times and then titrated using TCID50 (39). The passage 2 virus was used for another round of infection at MOI 0.1 for 8 hrs before RNA extraction using the QIAGEN RNeasy kit (QIAGEN, 74106).

ProtoScript II First Strand cDNA Synthesis Kit (New England Biolabs, E6560L) was used to generate first strand cDNA using a reverse primer in the 3’UTR, TGGTTATAACAAATTTACCCCCACCA. Then, the cDNA was used as a template in four independent Q5 PCR reactions with 25 cycles. Primers for amplifying the replication protein region were TCAAAGCCAACCCAAATTATGCT (forward) and TGGTTATAACAAATTTACCCCCACCA (reverse). Sequencing libraries for the input plasmid library were prepared as above, beginning with the PCR step. Gel purified amplicons were used as input for Illumina sequencing library preparation using the Twist Biosciences Enzymatic Fragmentation 2.0 kit with Universal Adapters (Twist Biosciences, 104207) with 180–220 bp target fragment sizes. Illumina libraries were pooled (6 samples per run) and sequenced with a NextSeq 2000 XLEAP P2 flow cell, 300 cycle paired-end kit (Illumina, 20100985).

### Sequencing Mutational Scanning Libraries

#### Sequence Analysis

DRAGEN BCL Convert (4.2.7) was used to demultiplex Illumina sequencing reads generating fastq files for the samples. Sequencing reads were mapped using minimap2 (minimap2/2.26) with the short read (sr) flag. Mapped reads were input into the GATK Analyze Saturation Mutagenesis tool (gatk/4.5.0.0) to identify codon changes from the wild-type reference sequence. A custom R script, codonFilter.r, was then used to filter for designed codon changes and convert the reads into hgvs format for use in Enrich2.

#### Enrich2 Analysis

To assess changes in variant frequency after selection (viral passage), we used Enrich2 with the scoring method set to Log Ratios (Enrich2) and normalization method set to Library Size (All Reads) (40). Relative enrichment, indicating how much more abundant a variant becomes under inhibitor selective pressure compared to the control condition, was calculated by subtracting the Enrich2 score for the control condition from the Enrich2 score for the inhibitor condition. The mean was computed for the three biological replicates, and this score was then squared to obtain the relative enrichment fold change. A fold change of 1 indicates no difference in variant abundance between the two conditions. The 99th percentile bootstrap enrichment value in the control conditions (enrichment score of 5) was used as the threshold to classify a mutation as enriched.

#### Structural analysis

The Chai-1 structure prediction framework (41) was used to model ligand interactions with viral proteins. **S**implified **M**olecular **I**nput **L**ine **E**ntry **S**ystem (SMILES) of ligands were obtained from pubchem. For prediction of Rupintrivir binding with the 3C(pro) wildtype or mutants, the SMILES of Rupintrivir, CCOC(=O)/C=C/[C@H](C[C@@H]1CCNC1=O)NC(=O)[C@H](CC2=CC=C(C=C2)F)CC(=O)[C@H](C(C)C)NC(=O)C3=NOC(=C3)C, was used as an input together with the amino acid sequence of the 3C(pro) variants. For the prediction of Guanidine hydrochloride binding with 2C(hel), the SMILES of Guanidine, C(=N)(N)N, and the SMILES of Hydrochloric Acid, Cl were inputed as two separate ligands together with the amino acid sequence of the 2C(hel).

The Boltz-1 (42) prediction model (version 0.4.0) was used to generate conformational ensembles of viral proteins and their corresponding mutants. The predictions used the multiple sequence alignment server (MMseqs2(43)) using 10 recycling steps and 200 diffusion samples. The step scale was reduced to 1 to increase the diversity among samples. The code used was the following boltz predict .fasta --use_msa_server --recycling_steps 10 --diffusion_samples 200 --output_format pdb --override --step_scale 1. Each conformational ensemble prediction was performed in three independent runs. The AlphaFold3 web server (44) was used to predict structures of wild type and mutant viral proteins and model their interaction with host factors. The 2A(pro) structure was predicted by inputting its corresponding amino acid sequence. Six copies of the 2C(hel) amino acid sequence were used as an input to predict the structure of the 2C(hel) hexamer. To predict hexameric 2C(hel) interaction with activated Arf1, the 2C amino acid sequence together with the Arf1 sequence (Uniprot, P84077) and the GTP ligand were used as input. The “morph” function in ChimeraX was used to create a trajectory visualizing the structural changes between the two atomic models of the 2C hexamer, with and without Arf1. The 3A dimer structure was predicted by inputting two copies of its corresponding amino acid sequence. To predict 3A dimer interaction with GBF1 or PI4KIIIβ and ACBD3, the 3A amino acid sequence together with the GBF1 (Uniprot, Q92538) or PI4KIIIβ (Uniprot, Q9UBF8) and ACBD3 (Uniprot, Q9H3P7) sequences were used as input.

The default “atomic distance” ≤ 3.5 Å option within the “Select Contacts” function of UCSF ChimeraX (45) was used to identify the contact residues between the viral and host proteins. The atoms of the contact residues are shown in sphere mode. For identifying contacts between the Fluorine atom at the P2 position of Rupintrivir and 3C protease residues, the “Contacts” function within “Structural Analysis” was used with the default Van Der Waals (VDW) overlap cutoff at −0.40 Å.

#### Statistics, reproducibility, and data analysis

The mutational scanning experiments were performed in three biological replicates. Statistical data analysis and visualization was performed using R (4.3.0). The *zoo* package was used to calculate the rolling mean metrics. The four-parameter log-logistic function in the *drc* package was used to model the data, allowing estimation of IC_50_, IC_90_, and IC_99_ concentrations that were used as selective pressures in the passaging experiments. *tidyverse* was used for dataframe manipulation, and plots were generated using a combination of *ggplot2* and *cowplot*. *coin* was used to perform a one-sided Wilcoxon-Mann-Whitney test to compare whether the distributions of max enrichment scores of the two groups (inside or outside contact sites) are statistically significant. The alternative hypothesis was that residues within contact sites are expected to have higher maximum enrichment scores compared to those outside contact sites. The *bio3d* package was used to handle PDB files and to calculate the Root Mean Square Fluctuation (RMSF) of the Cα carbons in a conformational ensemble.

## Supplementary Material

Supplement 1

## Data Availability

All code and analysis scripts used to generate the figures and analysis reported here will be included in a GitHub repository. All raw sequencing read data will be publicly available in the NCBI Short Read Archive.

## Highlights

- Replication organelles of positive-sense RNA viruses orchestrate genome replication through complex virus–host interactions
- Conventional deep mutational scanning cannot resolve molecular mechanisms of these organelles
- Combining mutational scanning, pharmacological perturbations, and structural modeling elucidates molecular features of the replication complex
- Reveals spatially segregated functional domains and distinct virus–host/inhibitor interfaces that define replication organelle architecture
