# Supplementary material for: Genotype-by-Inhibitor Interactions to Dissect Enterovirus Replication
Source: Res Sq. 2025 Sep 29:rs.3.rs-7660613. Preprint. [Version 1] doi: 10.21203/rs.3.rs-7660613/v1 (PMC12622148; doi:10.21203/rs.3.rs-7660613/v1)
Supplement: Supplement 1 [file NIHPPRS7660613v1-supplement-1.pdf]

SUPPLEMENTARY FIGURES

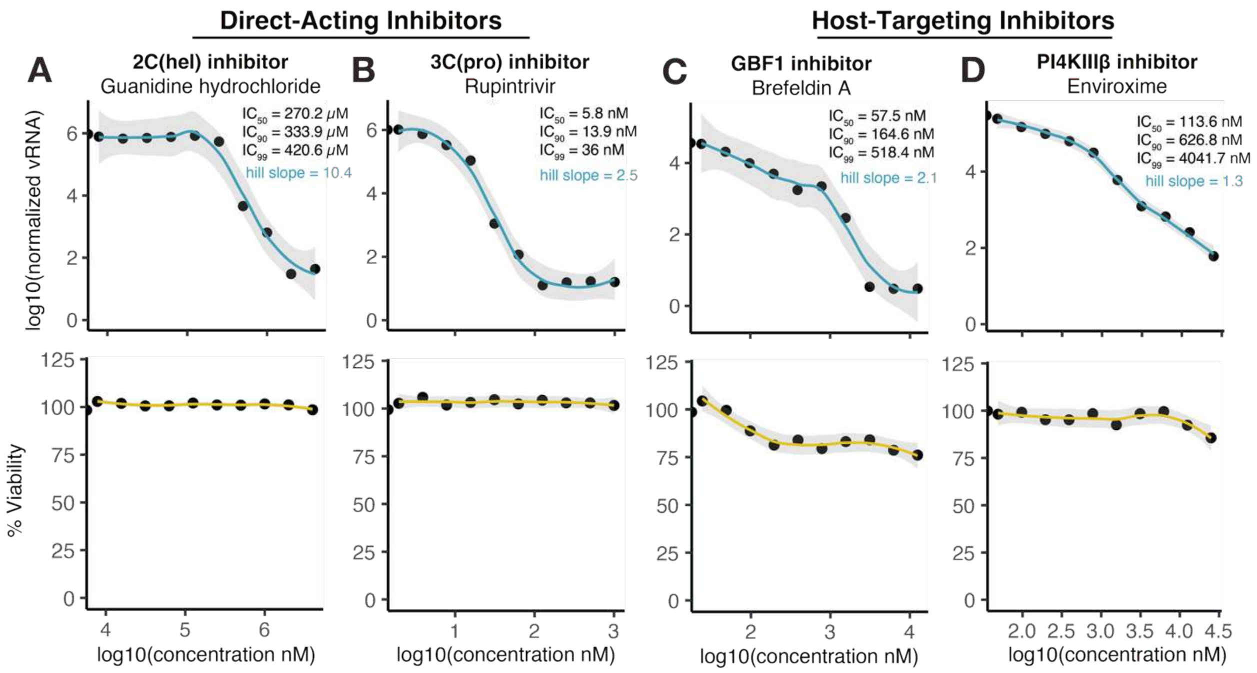

Supplementary Figure 1: Effect of Inhibitors on EV-A71 Replication and Cell Viability *Dose-response curves showing the effect of* ***(A)*** *Guanidine hydrochloride,* ***(B)*** *Rupintrivir,* ***(C)*** *Brefeldin A, and* ***(D)*** *Enviroxime on EV-A71 RNA levels (top panels) and cell viability (bottom panels).*

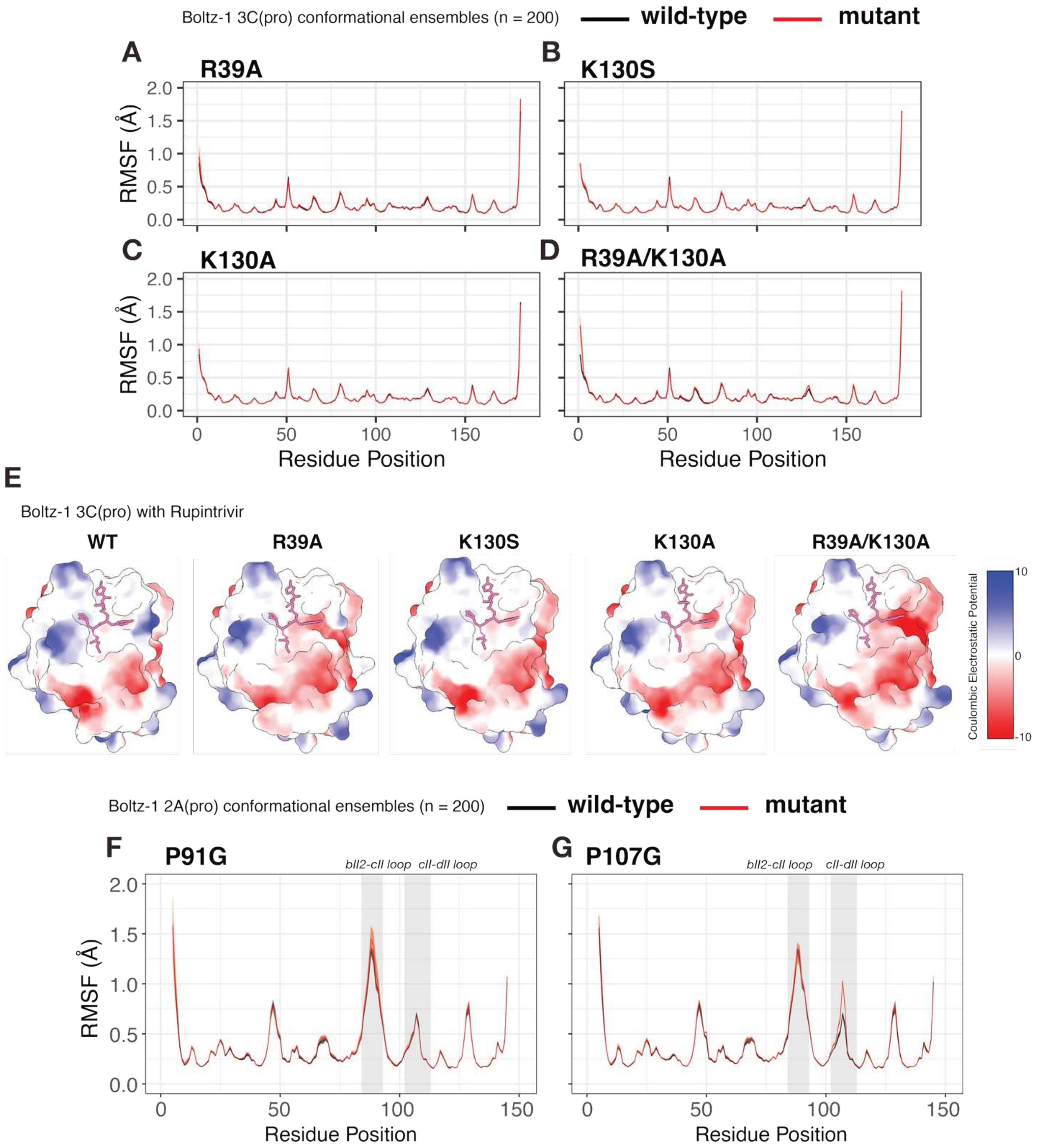

Supplementary Figure 2: Structural Impact of 2A and 3C protease Rupintrivir Enriched Mutations *Line plots showing the Root Mean Square Fluctuation (RMSF) in Ångströms (Å) for wild-type 3C protease (black line) compared to mutant 3C proteases (red line): **(A)** R39A, **(B)** K130S, **(C)** K130A, and **(D)** Double mutant (DM) with R39 and K130A mutations. The standard deviation is calculated from three independent runs and shown using geom_ribbon in ggplot2. **(E)** Structural models (Boltz-1) of the wild type and mutant forms of the 3C protease with Rupintrivir are shown. Surfaces are colored according to Coulombic electrostatic potential, with red indicating negative charge and blue indicating positive charge. Rupintrivir is shown in pink. Line plots showing the RMSF in Å for wild-type 2A protease (black line) compared to mutant 2A proteases (red line): **(F)** P91G and **(G)** P107G. The standard deviation is calculated from three independent runs and shown using geom_ribbon in ggplot2. The bII2-cII and cII-dII loops are highlighted with gray boxes.*

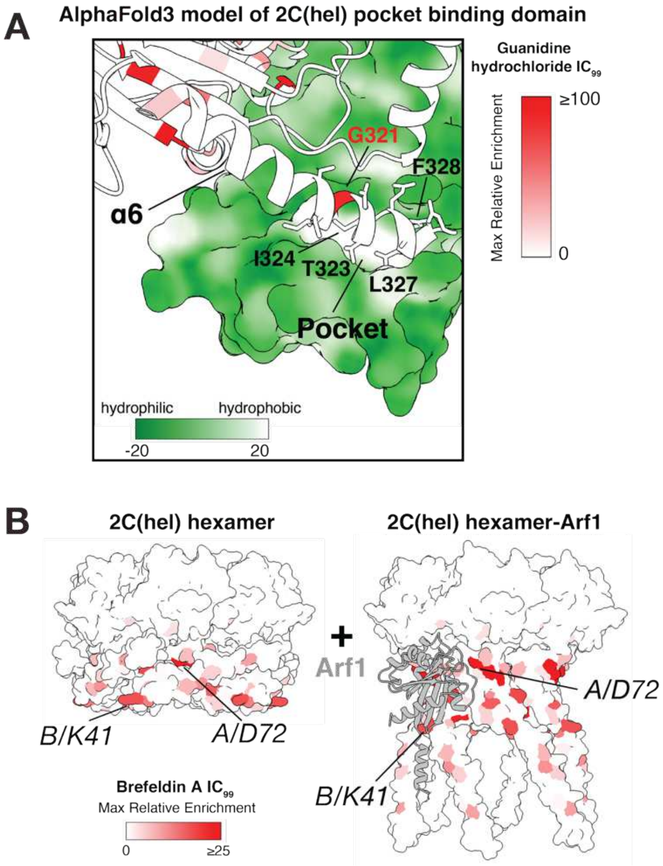

Supplementary Figure 3: **AlphaFold3 Models of the 2C hexamer and interaction with Arf1** ***(A)*** *Structural model showing the 2C multimer interactions. One 2C chain is colored according to the enrichment of mutations to Guanidine hydrochloride and the other chain (pocket) is colored by hydrophobicity.* ***(B)*** *Structural model showing the 2C multimer with or without Arf1. Annotated residues are in contact with Arf1 and overcome Brefeldin A inhibition***.**

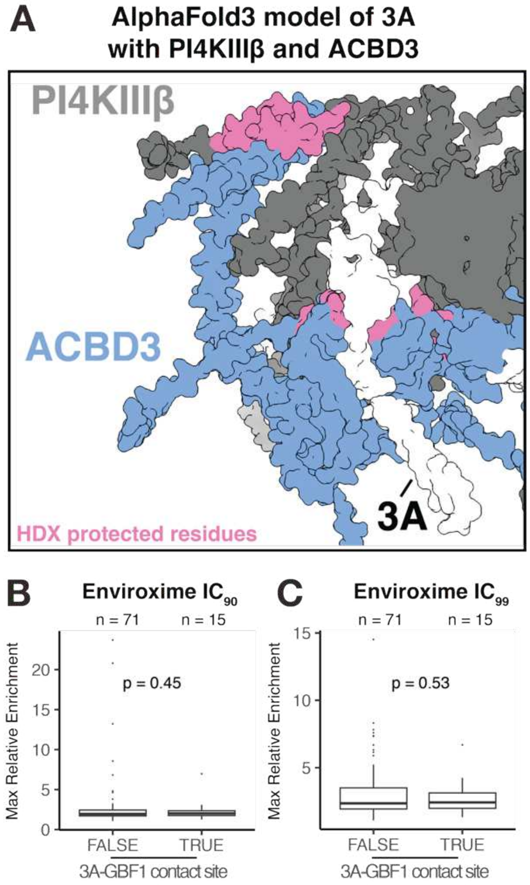

Supplementary Figure 4: Hydrogen/Deuterium Exchange Data Mapped onto the AlphaFold3 Model of the 3A-PI4KIIIβ-ACBD3 Complex and Enviroxime Enriched Mutants and GBF1 Contact Sites ***(A)*** *Residues protected from Hydrogen/Deuterium exchange are highlighted in pink. Data for highlighting residues was retrieved from McPhail et al.* (32) ***(B-C)*** *Box plot comparing the max relative enrichment of Enviroxime mutations at 3A residue position that interact (TRUE) or do not interact (FALSE) with GBF1. Statistical significance was determined using a one-sided Wilcoxon-Mann-Whitney test.*
